# Physically Crosslinked Chitosan/PVA Hydrogels Containing Honey and Allantoin with Long-Term Biocompatibility for Skin Wound Repair: An In Vitro and In Vivo Study

**DOI:** 10.3390/jfb12040061

**Published:** 2021-11-11

**Authors:** Mojtaba Koosha, Hadis Aalipour, Mohammad Javad Sarraf Shirazi, Ali Jebali, Hong Chi, Sepideh Hamedi, Nianxing Wang, Tianduo Li, Hamideh Moravvej

**Affiliations:** 1 Shandong Provincial Key Laboratory of Molecular Engineering, School of Chemistry and Chemical Engineering, Qilu University of Technology (Shandong Academy of Sciences), Jinan 250353, China; chihong@qlu.edu.cn; 2 Chemical and Polymer Engineering Group, Faculty of Engineering, Yazd University, Yazd 8915818411, Iran; hadis.alipour67@gmail.com (H.A.); jsarraf@yazd.ac.ir (M.J.S.S.); 3 Department of Laboratory Sciences, School of Paramedicine, Shahid Sadoughi University of Medical Sciences, Yazd 8916978477, Iran; alijebal2011@gmail.com; 4 Faculty of New Technologies Engineering, Shahid Beheshti University, Tehran 1983969411, Iran; se_hamedi@sbu.ac.ir; 5 Skin Research Center, Shahid Beheshti University of Medical Sciences, Tehran 1983963113, Iran

**Keywords:** wound dressing, freeze–thaw, chitosan, honey, allantoin, polyvinyl alcohol

## Abstract

Chitosan/PVA hydrogel films crosslinked by the freeze–thaw method and containing honey and allantoin were prepared for application as wound dressing materials. The effects of the freeze–thaw process and the addition of honey and allantoin on the swelling, the gel content and the mechanical properties of the samples were evaluated. The physicochemical properties of the samples, with and without the freeze–thaw process, were compared using FTIR, DSC and XRD. The results showed that the freeze–thaw process can increase the crystallinity and thermal stability of chitosan/PVA films. The freeze–thaw process increased the gel content but did not have a significant effect on the tensile strength. The presence of honey reduced the swelling and the tensile strength of the hydrogels due to hydrogen bonding interactions with PVA and chitosan chains. Long-term cell culture experiments using normal human dermal fibroblast (NHDF) cells showed that the hydrogels maintained their biocompatibility, and the cells showed extended morphology on the surface of the hydrogels for more than 30 days. The presence of honey significantly increased the biocompatibility of the hydrogels. The release of allantoin from the hydrogel was studied and, according to the Korsmeyer–Peppas and Weibull models, the mechanism was mainly diffusional. The results for the antimicrobial activity against *E. coli* and *S. aureus* bacteria showed that the allantoin-containing samples had a more remarkable antibacterial activity against *S. aureus*. According to the wound healing experiments, 98% of the wound area treated by the chitosan/PVA/honey hydrogel was closed, compared to 89% for the control. The results of this study suggest that the freeze–thaw process is a non-toxic crosslinking method for the preparation of chitosan/PVA hydrogels with long term biocompatibility that can be applied for wound healing and skin tissue engineering.

## 1. Introduction

Skin is the largest organ of the body and first line of defense against the external environment because of its dense surface and corneous layer [1,2]. A wound is an injury or tear that may be created on the skin through physical, chemical, mechanical and/or thermal damage. Healing of the injured skin is essential to protect the internal organs of the body from the entrance of external pathogenic agents. Wound healing includes biological and physiological stages of the healing process, including the migration and proliferation of a plethora of cell types in conjunction with the secretion of various growth factors and hormones [1,3]. Wound dressings are used for the protection of wounds from infection and also for acceleration of the healing process. A favorable wound dressing should have the following properties: (a) creation of a moist wound healing environment; (b) absorption of extra exudates secreted from the wound; (c) the ability to stimulate the tissue growth rate; (d) non-toxicity, antibacterial action and biocompatibility; (e) elasticity [4]. In burn and wound care, *Escherichia coli (E. coli)* and *Staphylococcus aureus (S. aureus)* are among the common bacteria that may enter and proliferate rapidly at the wound site, causing serious infections [5]. Preserving the wound from infection is a major concern in wound healing. The antimicrobial activity of a wound dressing is an advantage that can reduce the bacterial growth at the wound site. 

Hydrogels are 3D networks of hydrophilic polymers produced by chemical or physical crosslinking of water-soluble polymers. Hydrogels can hold large amounts of water in their structures without dissolution. Due to their special virtues, such as absorbing extra exudate, non-toxicity and biocompatibility, hydrogels are used as wound dressings [1,2,5,6]. Chitosan is a semi-crystalline macromolecular polysaccharide that is created from repeat units of N-acetyl glucosamine and glucosamine [7]. Chitosan has excellent biological properties, such as non-toxicity, biocompatibility and antibacterial and antifungal activity, favoring its use in many biomedical applications such as wound dressings, drug delivery systems and scaffolds for tissue engineering [6]. Chitosan is a well-known natural biopolymer for wound healing applications. It depolymerizes into N-acetyl-β-D-glucosamine, which initiates the proliferation of fibroblasts and accelerates collagen synthesis and deposition [8]. Polyvinyl alcohol (PVA) is a synthetic polymer synthesized from the hydrolysis of acetate groups of polyvinyl acetate. PVA is a water-soluble, non-toxic and biocompatible polymer. Its hydrophilic nature makes it very appropriate for blending with natural polymers such as chitosan in the preparation of wound dressings [2,9]. PVA or PVA blends can be crosslinked to form hydrogel networks. Physical or chemical methods may be used for PVA crosslinking. Freezing and thawing, irradiation and heat treatment are examples of physical methods. In chemical methods, a crosslinker reacts with the hydroxyl groups of PVA to form the network. The crosslinkers used are usually toxic and may affect the biocompatibility of the samples and react with drugs or other compounds present in the matrix. Physical methods are preferred when the biocompatibility of the hydrogel is essential. However, among the physical methods, heat treatment or irradiation can affect the drugs or biologically active compounds present in the wound dressing. Consequently, the freeze–thaw method is preferred because it provides the least damage to the structure of the matrix and the drugs or therapeutic compounds present in the wound dressing. It has been shown that the freeze–thaw gelation process forms crystalline regions in the PVA microstructure which act as junction points of the network. The degree of crystallinity and the size of the crystals increase with an increase in the number of freeze/thaw cycles and the temperature domain of the freezing/thawing process [10,11].

Allantoin is a compound formed by the oxidation of uric acid [12]. It is a tissue-promoting substance found in the root of comfrey. For a very long time, Europeans have used comfrey roots rich in allantoin for healing ulcers [13]. Nowadays, allantoin is mostly used in cosmetics. It is a common ingredient in many skin care formulations. It has analgesic activity and has been used for wound healing, skin hydration, removal of necrotic tissue and prevention of gastric ulcers [14,15]. Allantoin is a safe, non-toxic compound and has tissue repair properties. It is also found in the excretions of maggots in maggot debridement therapy and has been reported to directly stimulate the healing process [16].

Honey has special biological and physical properties beneficial for all stages of wound healing [17]. The biological properties are antibacterial activity, immune stimulation and anti-inflammatory and debriding action. The physical properties of honey are high bio-adhesion, high viscosity and low pH near 3.2–4.5. The high viscosity, low pH and high amounts of sugar in honey prevent the growth of microbes [18,19]. In recent years, honey has been incorporated into many wound dressings based on synthetic or natural polymers, and its beneficial effects on wound healing has been noted [10,20,21,22,23,24]. In a previous study, hydrogel sheets of chitosan/gelatin and honey were applied as wound dressing materials; however, none of the chemical or physical crosslinking methods were used for preparation of the hydrogels [22]. In another study, a combination of irradiation and the freeze–thaw method was used for crosslinking PVA/carboxymethyl chitosan/honey hydrogels [25]. Although the mechanical strength of the hydrogels was improved, it was mentioned that the combination method significantly reduced the swelling capacity of the hydrogels, which limits the absorbance of wound exudates by the wound dressing. Irradiation also degrades the chemical structure of polymers [26,27] and denatures the structure of proteins or other biopolymers [28,29]. In another study, Manuka and Indonesian honeys were compared for supporting the application of a plasma jet during the proliferative phase of wound healing [30]. In addition, Manuka honey was mixed with chitosan to prepare bioactive wound dressings that were more effective than commercial dressings [31]. In a recent publication, a low-cost, antibacterial and ecofriendly membrane was synthesized from PVA, chitosan and honey and introduced as a potential candidate for wound dressing applications [32]. The authors did not test the biocompatibility or wound healing ability of the membranes. In previous research, the biocompatibility of hydrogels was evaluated routinely over a short period (1–7 days). We believe that in order to prove the safety of the freeze–thaw method for crosslinking PVA/chitosan hydrogels, a long-term biocompatibility study should be performed, and this is the aim of the present study. Furthermore, the preparation, characterization and properties of freeze–thawed chitosan/PVA hydrogels in the presence or absence of honey and/or allantoin have not been considered previously in the literature.

In the present study, hydrogel sheets based on chitosan/PVA containing honey and allantoin were prepared for application as tissue engineering and wound dressing materials. Chitosan was used due to its beneficial effects on wound healing. It was blended with PVA to improve the mechanical properties of the hydrogels. Honey was blended with the films to improve the antibacterial properties and allantoin was used to induce tissue repair and stimulate wound healing. The freeze–thaw method was selected for crosslinking the samples in order to prepare highly biocompatible hydrogels, preserve the biological properties of honey and avoid the adverse effects of chemical crosslinking. To the best of our knowledge, studies on freeze–thawed chitosan/PVA hydrogels containing honey and allantoin have not been reported in the literature up to the present time. Furthermore, the long-term in vitro biocompatibility of freeze–thawed hydrogels has not been evaluated previously. In this study, we evaluated the long-term biocompatibility of the hydrogels via an MTT assay. The swelling capacities and mechanical properties of the hydrogel wound dressings were measured. The antibacterial activity of the wound dressings was tested against *Escherichia coli (E. coli)* and *Staphylococcus aureus (S. aureus).* Finally, the wound dressings were tested in vivo using Wistar rats as animal models, and the wound closure was observed.

## 2. Materials and Methods

### 2.1. Materials

Chitosan (low molecular weight MW = 50,000–190,000 Da, viscosity (Brookfield, 1% in 1% acetic acid at 25 °C = 2–300 cPs) 75–80% deacetylated), PVA (M_n_ = 146,000–186,000 g/mol, degree of hydrolysis 99%) and allantoin (purity ≥ 98%, melting point 230 °C) were purchased from Sigma-Aldrich (St. Louis, MO, USA). Honey collected from agricultural farms in the countryside of Yazd Province was purchased from a local herbal medicine store (Kordiha herbal store, Yazd Province, Yazd, Iran). The honey used was a common type of honey. Glacial acetic acid and phosphate-buffered saline (PBS) were purchased from Merck (Darmstadt, Germany). Double-distilled water was used as a solvent for solution preparation.

### 2.2. Preparation of the Hydrogels

A 1.5% (*w/v*) solution of chitosan was prepared by dissolving 1.5 g of chitosan into 98.5 mL of 0.5% (*v/v*) acetic acid (pH ≅ 2.85) by vigorous stirring at RT until dissolution was complete. Ten grams of PVA powder was gradually added into 90 mL of distilled water at 80 °C to prepare a 10% (*w/v*) solution by magnetic stirring. The chitosan and PVA solutions were mixed at a ratio of 30/70 (*v/v*). The honey was diluted with the distilled water in a 1:1 (*w/w*) ratio (pH ≅ 4.0 after dilution). Allantoin was added into the distilled water at 80 °C with a concentration of 4% and stirred until completely dissolved. Four different samples were prepared, with and without honey and allantoin. The chitosan/PVA solution was blended with predetermined volumes of allantoin and honey solutions in such a way that the final mass % of honey and allantoin in the films was according to Table 1. The solutions were mixed by magnetic stirring for 1 h then casted into petri dishes. The petri dishes had a diameter of 8 cm and were gently filled with ~30 mL of each solution. To prepare the hydrogel films, the solutions were crosslinked by freezing to −20 °C for 18 h and thawing to 25 °C for 6 h. Three freezing/thawing cycles were applied for each sample. Finally, the solutions were casted for 24 h to remove the solvent. 

### 2.3. Macroscopic Visualization and Colorimetric Analysis

In order to visualize the samples, macroscopic images were taken from the hydrogel films during and after preparation. The images were captured during the freeze–thaw cycles before and after freezing and from the final hydrogels in the dry and swollen states. In addition, the colorimetric parameters of the dry hydrogel films including L* (lightness), a* (red–green) and b* (yellow–blue) were measured using a colorimeter (DRK103B brightness and color tester, Drick instruments, China). Black and white standard plates were used for calibration. The yellowness index (*YI*) and the whiteness index (*WI*) were calculated using Equations (1) and (2), respectively.

(1) $$YI=142.86\;\left(\frac{b^{*}}{L^{*}}\right)$$

(2) $$WI=100-\sqrt{{\left(100-L^{*}\right)}^{2}+a^{{*}^{2}}+b^{{*}^{2}}}$$

### 2.4. Characterization

In order to better understand the effect of the freeze–thaw treatment on the physicochemical properties of the hydrogels, they were characterized using attenuated total reflectance Fourier transform infrared spectroscopy (ATR-FTIR) (Tensor 27, Bruker, Karlsruhe, Germany) and X-ray diffraction (XRD) (LabX XRD-6100, Shimadzu, Kyoto, Japan). For ATR-FTIR, the surfaces of the samples were cleaned and placed under the ATR apparatus, and the spectra were collected. XRD patterns were obtained using the device with a radiation source of CuKα which has a wavelength of 1.5406 Å, corresponding to an energy of 8.04 keV. The samples were scanned in the range 2θ = 5–40° with a step size of 0.05°.

### 2.5. Thermal Behavior

The thermal behavior of the samples was measured using differential scanning calorimetry (DSC). DSC thermograms were recorded using a thermal analysis device (DSC Q10, TA Instruments, New Castle, DE, USA) from room temperature to 300 °C with a heating rate of 10 °C/min under a nitrogen gas atmosphere.

### 2.6. Swelling Ratio

Before testing the swelling ratio, in order to remove previously absorbed moisture in the hydrogels, the samples (with 3 replicates for each sample) were cut (2 cm × 2 cm) and dried under vacuum at 60 °C for 12 h. The swelling ratio was measured gravimetrically in PBS (pH = 7.4) at 37 °C after 24 h. The swelling degree was calculated according to Equation (3) [33]:

(3) $$\mathrm{Swelling}\;\mathrm{Ratio}\;(\%)=\frac{W_{s}-W_{d}}{W_{d}}\;\times \;100$$

where *W_s_* is the weight of the swollen hydrogel and *W_d_* is the weight of the dry hydrogel.

### 2.7. Gel Content

The gel content values of the samples with and without the freeze–thaw treatment were measured according to methods previously described in the literature. The samples were cut into small pieces, wrapped with filter paper and weighed (*W*_1_). Each sample was soaked in boiling water at a temperature of 100 ± 1.0 °C for 24 h. Then, the samples were dried in a vacuum oven at a temperature of 50 ± 0.1°C and weighed again (*W*_2_). The gel content of the samples was calculated gravimetrically according to Equation (4). Three replicates were tested for each sample, and the data were averaged.

(4) $$\mathrm{Gel}\;\mathrm{Content}\;(\%)=\frac{W_{1}-W_{2}}{W_{2}}\;\times \;100$$

### 2.8. In Vitro Degradation 

The in vitro degradation of the samples was measured by placing pre-weighed samples in 1.5 mL microtubes filled with PBS. The microtubes were incubated at 37 ± 0.1 °C in a shaking incubator (Pars Azma Company, Tehran, Iran) for 16 days. After this time, the PBS was removed, and the samples were dried until they reached a constant weight. The mass loss was calculated according to Equation (5):

(5) $$\mathrm{Mass}\;\mathrm{Loss}\;(\%)\;=\;\frac{W_{1}-W_{0}}{W_{0}}\;\times \;100$$

where *W*_0_ is the initial weight of the sample and *W*_1_ is the weight of the sample after drying.

### 2.9. Mechanical Properties

The mechanical properties of the hydrogels in the dry state were measured according to the standard method of ISO 37. The films were cut into dumbbell shapes with a length of 25 mm and a width of 6 mm. All the films had a thickness of about 0.35 mm. The samples were mounted into the grips with a gauge length of 25 mm and stretched using a tensile tester (SDL micro 350, Testometric, Rochdale, UK) equipped with a load cell of 500 N at a strain rate of 50 mm/min, until breakage. The tensile strength and the elongation at breaking for each sample were expressed in terms of the average ± standard deviation by testing and averaging at least 3 replicates.

### 2.10. Allantoin Release Measurement

In order to plot the calibration curve for measuring allantoin concentration, allantoin was dissolved in PBS of different concentrations from 10–50 μg/mL and the maximum absorption at a wavelength of 204 nm was obtained using a UV-VIS spectrophotometer (Optizen POP, Daejeon, South Korea). The concentration of allantoin in the solution was related to the absorption using Beer–Lambert’s law. The release of allantoin was measured for the hydrogel sample H3. The hydrogel film was cut into a rectangular shape with dimensions of 2 cm × 2 cm and placed in 25 mL of PBS at 37 °C under stirring. The absorption was measured at different time points by taking out a volume of 5 mL from the release medium and replacing it with 5 mL of fresh PBS, to maintain a constant release volume. The concentration at each time point was measured according to the calibration curve and further modified using Equation (6) to eliminate the effect of added PBS after each sampling [34]:

(6) $$C_{r}=C_{n}+\frac{C_{n-1}\times V_{s}}{V_{t}}\;$$

where *C_r_* is the corrected concentration of the nth sample, *C_n_* is the measured concentration of allantoin in the nth sample, *C_n−_*_1_ is the measured concentration of the (*n*−1)th sample, *V_t_* is the volume of the release medium (25 mL) and *V_s_* is the volume of the sample drawn off (5 mL).

In order to study the kinetics of the release of allantoin from the hydrogel, the following models (Equations (7)–(9)) were fitted to the release data using the MATLAB curve fitting toolbox:

(7) $$\frac{M_{t}}{M_{0}}=K_{KP}t^{n}\;\mathrm{Korsmeyer}\text{–}\mathrm{Peppas}\;\mathrm{model}$$

(8) $$\frac{M_{t}}{M_{0}}=K_{H}t^{0.5}\;\mathrm{Higuchi}\;\mathrm{model}$$

(9) $$\frac{M_{t}}{M_{0}}=1-e^{\frac{-{\left(t-T\right)}^{b}}{a}}\;\mathrm{Weibull}\;\mathrm{model}$$

In the above models, *M_t_* is the amount of drug released at time t and *M_0_* is the amount of drug initially loaded into the hydrogel. In Equation (7), *K_KP_* is the Korsmeyer-Peppas or velocity constant which is related to the structural and geometrical characteristics of the system and n is the exponent of release which is related to the drug-release mechanism. In Equation (8), *K_H_* is the release constant of the Higuchi model which is mainly related to the diffusion coefficient and the solubility of the drug in the matrix medium. In Equation (9), a is a scale parameter which defines the timescale of the process, *b* characterizes the type of curve (*b* = 1 for exponential, *b* > 1 for sigmoid and *b* < 1 for parabolic) and *T* is the localization parameter, which is taken here as 0 since the release starts readily by immersing the sample in an aqueous medium.

### 2.11. Cytocompatibility

To evaluate the biocompatibility of the samples, the indirect MTT assay method using extracts of the samples in a culture medium was performed, based on our previous work [35]. The samples (with 6 replicates for each sample) were sterilized via electron beam irradiation, cut into rectangular shapes with dimensions of 2 cm × 1 cm and placed in 70% ethanol (containing 30% of 0.1 M NaOH) for 30 min for the removal of the remaining acetic acid. The samples were washed 3 times with sterilized PBS for 10 min. Each sample was placed in 2 mL of serum-free culture medium (DMEM containing 1 vol.% of penicillin and streptomycin as antibiotics and 1 vol.% of L-glutamine) at 37 °C for 1, 4, 8, 23, 26 and 30 days. The control was the serum-free culture medium without a sample, incubated for similar time periods. The normal human dermal fibroblast (NHDF) cells (isolated from a human neck skin biopsy) were used for cytotoxicity studies. The cells were cultured in a 96-well plate at a density of 1 × 10^4^ cells per well, with each well containing 90 µL of DMEM and 10 µL of fetal bovine serum (FBS). The plate was incubated for 24 h at 37 °C in a CO_2_ incubator. After addition of the cells, the medium in each well was replaced by 90 µL of the extracts of the samples from the culture medium as well as from the control medium. In addition, 10 µL of FBS was added to each well. The plate was incubated for another 24 h and then the medium in each cell was replaced with 100 µL of MTT solution (0.5 mg mL^−1^ in PBS). The plate was incubated for a further 4 h. The MTT solution was removed and 100 µL of dimethyl sulfoxide (DMSO) was added to each well and incubated for 15 min with shaking. The absorbance at 570 nm was determined using an ELISA microplate reader (Model 680, Bio-Rad Laboratories, CA, USA). The biocompatibility values of the samples were reported as the % cell viability obtained by dividing the absorbance of each sample by the absorbance of the relevant control sample at each time point.

### 2.12. Wound Closure

An in vivo study using Wistar rats as animal models was performed to evaluate the effect of the hydrogel sheets on wound closure. Sample H2 was chosen for the animal study for two reasons: (1) it showed higher biocompatibility and (2) the presence of allantoin reduces the adhesion of the hydrogel to the wound (unpublished clinical data). All the procedures applied to the animals were approved by the Ethics Committee at Shahid Beheshti University of Medical Sciences (IR.SBMU.MSP.REC.1399.181). The ethics committee followed the guidelines according to the instructions and regulations for research in biomedical studies issued by the Iran National Committee for Ethics in Biomedical Research (Ministry of Health and Medical Education of the Islamic Republic of Iran) (ethics.research.ac.ir). A total number of 6 male Wistar rats (200–250 g) were provided by the Pasteur Institute of Iran and randomly divided into 2 groups. Diethyl ether was used for anesthetizing the rats. The back of the neck of each rat was shaved and one second-degree burn was created on each rat. A rectangular metal plate with an area of 3 cm^2^ (2 cm × 1.5 cm) was heated to 80 °C and rested on the skin of the rat for 10 s [36,37]. Group 1 (the control sample) received no treatment and the wounded area was only washed with normal saline. In group 2, a sheet of the H2 sample sterilized by electron beam irradiation (25 KGy) was cut with dimensions of 1.5 cm × 1 cm, swollen in sterile normal saline to reach the dimensions of the wound and carefully placed on the wound site of each rat. The hydrogel was replaced with freshly swollen samples every 24 h. The wound closure results were obtained from the images of the wounds captured on days 2, 7, 14 and 21. The percentage of wound closure was determined using Equation (10) [22]:

(10) $$C\;(\%)=\;\frac{A_{i}-A_{t}}{A_{i}}\;\times 100$$

where *A_i_* is the initial area of the wound and *A_t_* is the area of the wound at time *t*. The areas were measured using ImageJ software (ImageJ bundled with Java 1.8.0_172, National Institute of Health, New York, NY, USA).

### 2.13. Antibacterial Activity Test

The antibacterial activity of the hydrogels was tested against both Gram negative (*Escherichia coli*) and Gram positive (*Staphylococcus aureus*) bacteria according to the standard AATCC 100 (2004). A bacterial suspension was prepared in a nutrient broth (NB) by adjusting its turbidity to 0.5 McFarland standard. The samples were cut into 1 cm × 1 cm squares and disinfected using 70% ethanol prior to irradiation with a UV lamp for 20 min. Each sample was placed at the bottom of a sterile tube and 1 mL of the bacterial suspension was poured into it. One tube containing bacterial suspension without the sample was used as a control. All tubes were incubated for 24 h at 37 °C. Then, 100 µL of each inoculum was serially diluted and cultured in the plates prefilled with nutrient agar. The plates were incubated for 24 h at 37 °C. The number of colonies grown on each plate was counted and compared to the control [38]. Finally, the antibacterial efficiency was calculated based on Equation (11):

(11) $$E\;\left(\%\right)=\left(\frac{A-B}{A}\right)\times 100$$

where *A* is the number of colonies grown from the control tube and *B* is the number of colonies grown from the tube containing the composite film.

### 2.14. Statistical Analysis

All the data with replications were statistically analyzed. A one-way analysis of variance (ANOVA) with a post hoc test using Tukey’s method was used for comparison between the means. The data were processed using Minitab software, and *p*-values of less than 0.05 were considered to indicate a significant difference.

## 3. Results and Discussion

### 3.1. Macroscopic Visualization and Colorimetric Analysis of the Hydrogels

**Figure 1:**
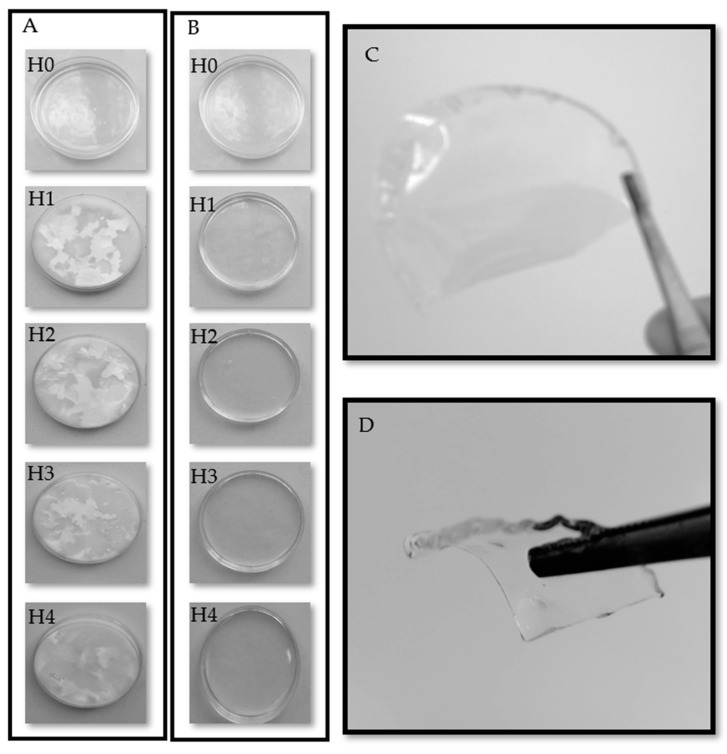
Macroscopic images from the hydrogels during preparation (**A**) after freezing and (**B**) after thawing, with the final product (**C**) in the dry state and (**D**) in the swollen state.

Macroscopic images were obtained from the hydrogels during preparation, especially when the freeze–thaw method was used for crosslinking. Figure 1A presents the images of the hydrogels after freezing. Except for sample H0 which was not frozen, crystalline regions are observed for all the other samples after freezing. As mentioned in the Introduction, the freeze–thaw method can induce crystalline regions in the structure of PVA. The crystalline regions are observable when the solutions are frozen, and they disappear after thawing (Figure 1B). The crystallinity of the final hydrogel can be detected by characterization methods such as XRD, as discussed in the next section. The final product of this research is a dry hydrogel film (Figure 1C). The films are flexible and after placing in water they swell in all dimensions (Figure 1D). 

Table 2 represents the results of the colorimetric analysis of the films in the dry state. As can be seen, the lightness (L*) of the chitosan/PVA films did not change significantly after the freeze–thawing process (H0 and H1 samples) but decreased after the addition of honey (H2 and H4 samples). The parameter b* showed an increase in the intensity of the yellow color of honey-containing films compared to chitosan/PVA films. The YI increase in films containing allantoin and honey (H2, H3 and H4) also confirmed the yellowness increment. The yellowness enhancement of the nanocomposite film is attributed to the yellowish tint of honey and allantoin as additives. Honey showed a stronger effect on yellowness than allantoin.

### 3.2. Structural Characterization of the Hydrogels

In order to determine the effects of the freeze–thaw process and the interactions between the components in the hydrogels, the samples were characterized by FTIR, XRD and DSC. 

**Figure 2:**
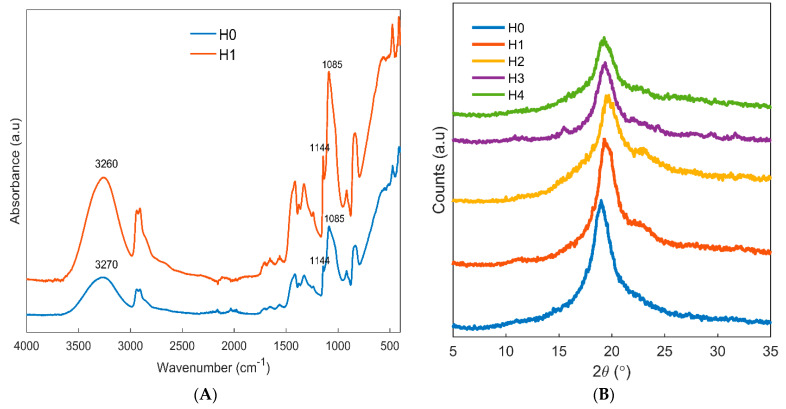
(**A**) FTIR spectra of freeze–thawed and non-freeze–thawed samples and (**B**) XRD patterns of the samples.

The FTIR spectrum of chitosan/PVA samples with and without the freeze–thaw treatment was obtained to monitor the changes induced by the freeze–thaw method in the chemical structure of the films. Both the samples showed the characteristic peaks related to the chemical groups of chitosan and PVA (Figure 2A). The band at 3270 cm^−1^ for the sample without the freeze–thaw treatment (H0) is related to the vibrations of the N-H and -OH groups of chitosan, as well as the hydroxyl groups of PVA. One strong peak in this region indicates favorable hydrogen bonding interactions between chitosan and PVA in the blend. After applying the freeze–thaw process (H1), this peak was shifted to 3260 cm^−1^, indicating that these groups need higher energy for their vibrational motions due to stronger interactions. The two peaks observed at 2940 and 2900 cm^−1^ for both samples are related to the stretching vibrations of the CH2 groups. Other peaks observed in the spectra of both samples are characteristic peaks of the chemical groups present in chitosan and PVA. The peaks at 1412 and 1375 are related to CH bending vibrations in the CH2 and CH3 groups. The peak at 1144 cm^−1^ can be assigned to C-C vibrations. The peak at 1085 cm^−1^ is related to the overlapping C-O stretching vibrations of chitosan and PVA. These two peaks are crystallinity-sensitive bands of PVA, in which the ratio of the absorbances can be used to calculate the degree of crystallinity of PVA according to a formula published in the literature [39]. According to this method, the degree of crystallinity was determined for the H0 and H1 samples using the ratio A1144/A1085. It was found that the degree of crystallinity increased from 38% for the non-freeze–thawed H0 sample to 42% for the freeze–thawed H1 sample. This result, in accordance with previous reports [10], indicates that the freeze–thaw process can increase the crystallinity of PVA/chitosan samples, as discussed further when considering the XRD results.

The XRD patterns of the non-freeze–thawed and freeze–thawed samples can provide valuable information about the crystallinity of the samples caused by the freeze–thaw process. In the pattern observed for H0 (Figure 2B), one crystalline peak was observed centered at 2θ~19°. After applying the freeze–thaw process, this peak was slightly shifted to higher values and a shoulder appeared at around 2θ~22°. This result indicated the formation of new crystalline areas in the structure of PVA/chitosan after applying the freeze–thaw process. This finding was in agreement with the degree of crystallinity of the samples calculated by the FTIR spectra. The sample containing honey (H2) did not show significant changes compared with the H1 sample. For the allantoin-containing sample, a small peak appeared at 2θ~15°, which may be related to the crystalline forms of allantoin. This peak is also present in the sample containing both honey and allantoin (H4) but with a lower intensity.

### 3.3. Thermal Behavior

**Figure 3:**
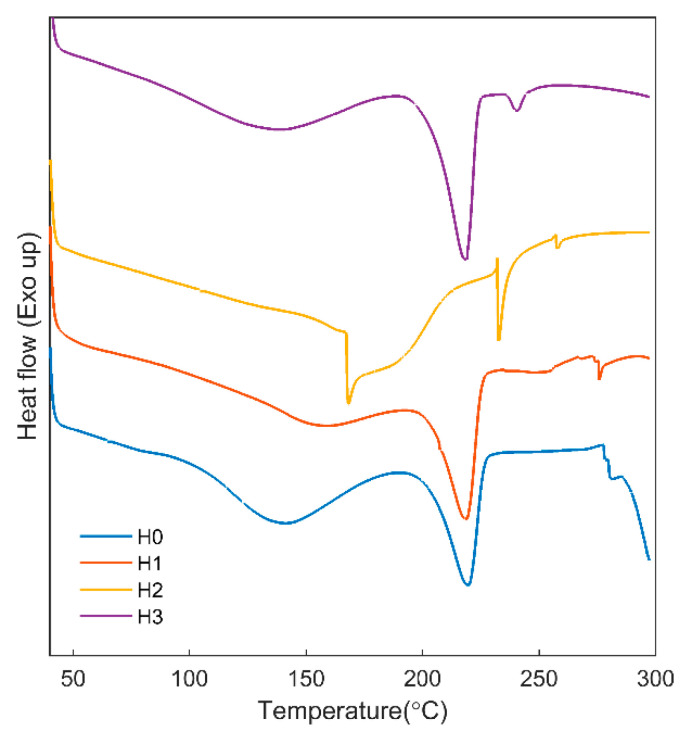
DSC thermograms of the samples at a heating rate of 10 °C/min under N_2_ atmosphere.

The DSC thermograms of the chitosan/PVA film without the freeze–thaw treatment (H0) show the typical thermal behavior of chitosan/PVA films (Figure 3). A small change in the baseline around 75 °C is related to the glass transition temperature of PVA. The broad endothermic peak centered at around 120 °C is due to the evaporation of water and acetic acid molecules bound in the microstructure of the PVA and chitosan chains. Melting of the H0 film occurred at 220 °C. This result indicates the compatibility between chitosan and PVA chains due to hydrogen bonding interactions. The degradation of the chitosan chains begins at around 275 °C, with an exothermic decline in the thermogram. After applying the freeze–thaw process to the solutions, the glass transition peak is diminished, which indicates the formation of physical crosslinks between the chains. The melting point did not show a significant change between the freeze–thawed and non-freeze–thawed samples. The H1 sample did not show a degradation peak. This result indicates that the freeze–thaw process increased the thermal stability of the chitosan/PVA film (H0). For the chitosan/PVA films containing allantoin (H3), a small endothermic peak was observed between 235–245 °C, which is related to the melting of allantoin crystals. In the case of the chitosan/PVA film containing honey (H2), the thermal behavior was changed significantly. Three endothermic peaks were observed at 168, 232 and 258 °C. The peak at 168 °C may be due to the melting of sugar crystals such as glucose, fructose and maltose. The peak at 232 °C may be related to the melting of PVA crystals; it is shifted to higher temperatures because of the strong hydrogen bonding interactions with honey. The peak at 258 °C may be due to the melting of other components of honey, such as minerals, carbohydrates or enzymes.

### 3.4. Swelling, Gel Content, Mass Loss and Mechanical Properties

The ability of a hydrogel to absorb exudates from a wound is important for its application as wound dressing. The swelling properties of the freeze/thawed hydrogel wound dressings are presented in Table 3. The swelling ratio could not be measured for the H0 sample because after 24 h it lost its mechanical properties, and the weight of the sample could not be measured. The chitosan/PVA cryogel (H1) showed a swelling ratio of 476 ± 24% in accordance with the literature data [40]. The addition of honey (H2) significantly reduced the swelling ratio to 421 ± 11%. A similar result was observed in our previous work on thermally crosslinked PVA/honey hydrogels [23]. Honey is a complex of mono/polysaccharides containing hydroxyl groups. These can form hydrogen bonds with the hydroxyl groups of PVA chains. When water molecules penetrate into the structure of the hydrogel, honey molecules also form hydrogen bonds with the H_2_O molecules. It is obvious that honey molecules are more water soluble than chitosan and PVA chains. Thus, they enhance the penetration of water molecules into the hydrogel and diffuse out of the network during the swelling measurement period. They also result in higher solubility of chitosan and PVA chains through hydrogen bonding with both water molecules and chitosan/PVA chains. Therefore, the presence of water-soluble honey molecules inside the structure of the chitosan/PVA hydrogel results in a lower swelling ratio. The addition of allantoin (H3) did not affect the swelling ratio significantly. However, the swelling ratio was significantly decreased to 324 ± 18% when both honey and allantoin (H4) were present in the hydrogels. Allantoin contains amine groups in its chemical structure which can form hydrogen bonds with chitosan/PVA chains, honey and water molecules. When it is included in the hydrogel network, it can form hydrogen bonds with honey and diffuse out of the network. Finally, when both honey and allantoin are present in the structure, they can have a synergistic effect in reducing the swelling of the hydrogel.

The gel content of the samples was measured at 100 °C after 24 h (Table 3). All the samples showed gel contents of less than 10%. The results show that the major parts of the samples were dissolved during this period at the temperature of the test. This result indicates that the nature of the crosslinking caused by the freeze–thaw process in chitosan/PVA hydrogel films is mainly physical, rather than chemical. Chemical crosslinks are more stable at this temperature and result in higher gel content values, while physical crosslinks, which are caused by hydrogen bonding interactions or the higher crystallinity of PVA, are more sensitive to temperature and result in lower gel content values.

The in vitro degradation of the hydrogels is important for biomedical applications. In this study, the mass loss of the samples at 37 °C in PBS was measured and reported (Table 3). For the non-crosslinked hydrogel (H0), the mass loss was 25.3 ± 5.9% after 16 days, while the freeze–thaw crosslinked sample (H1) showed a mass loss of only 12.6 ± 1.7%. The samples containing honey (H2 and H4) showed a higher mass loss, but sample H3 which contained allantoin only showed a mass loss of 36.7 ± 9.1%. The presence of honey increased the mass loss of the samples because it contains saccharides with low molecular weight that dissolve more easily in water compared to chitosan and PVA long-chain macromolecules. In addition, allantoin molecules have a lower molecular weight and are partially soluble in water. Removal of the small molecules results in a higher penetration of water molecules into the structure of the hydrogels and increases the mass loss.

The mechanical properties of the hydrogels are important for wound dressing materials. It is favorable for the tensile strength of a wound dressing to be close to that of natural, healthy skin. When stretching, it should not easily break. The mechanical properties of the prepared hydrogels were tested using tensile testing. The tensile strengths and elongations at breaking of the samples are given in Table 3. All samples showed suitable mechanical properties for soft tissue repair. It has been shown that the mechanical properties of human skin can change depending on the loading direction, the location of the skin and the test speed. The tensile strength of human skin is reported to vary from 7 to 40 MPa, with an average of 21.6 ± 8.4 MPa at a strain rate of 50 mm/min [41]. The values obtained for the tensile strength of the samples were close to those of human skin. The sample H0 showed the highest and H4 showed the lowest tensile strength; however, the tensile strength differences between the samples were not statistically significant (*p*-value 0.07~0.97). 

Comparing the H0 and H1 samples, it was found that the tensile strength and elongation at breaking were both decreased for the H1 sample, which was crosslinked via the freeze–thaw process. This result can be related to the increased crystallinity of the H1 sample after applying the freeze–thaw process. Among the samples, the H2 and H4 films containing honey showed the lowest strength and the highest elongation at breaking. In fact, the presence of honey in the samples reduces the modulus and increases the elongation. Honey is a mixture of mono, di and polysaccharides. It can act as a plasticizer in chitosan/PVA films, reducing the strength and increasing the elongation at breaking. Our result is in accordance with previously reported data [22].

### 3.5. Antibacterial Efficiency

It is favorable for a wound dressing to be able to prevent the growth of the bacteria which usually contaminate the wound and result in wound infection. In this study, the antibacterial efficiencies of the hydrogels were tested against *E. coli* and *S. aureus* (Table 3). As the data show, for both the Gram positive and Gram negative bacteria, all the hydrogels showed effective antibacterial activity. By comparing the results for *E. coli*, it can be observed that the antibacterial efficiencies of the samples H2 (82.00 ± 4.88), H3 (85.03 ± 5.36) and H4 (88.74 ± 5.97) were much higher than those of the H0 (13.20 ± 1.4) and H1(15.27 ± 2.19) hydrogels. Since the latter samples contained honey, allantoin or both, it can be concluded that the presence of honey and allantoin significantly increased the antibacterial efficiency of the samples. A similar conclusion can be drawn for the results for *S. aureus*. Furthermore, the antibacterial efficiencies of H3 and H4 were higher than those of the other samples. This result indicates that the presence of allantoin dramatically increased the antibacterial efficiency of chitosan/PVA hydrogels against *S. aureus*. Allantoin showed outstanding antibacterial activity against both selected bacteria. Recently, it was reported that the antibacterial activity of chitosan/gelatin/allantoin films was reduced for *S. aureus* compared to *E. coli*, due to higher amounts of peptidoglycan in the cell membrane of *S. aureus* [42]. The results of this study show that allantoin restricts the growth of *E. coli* bacteria more effectively than *S. aureus*. Lipopolysaccharides, which are present in the outer membrane of bacteria, can form hydrogen bonding interactions with amide groups present on the surface of allantoin crystals, resulting in lipopolysaccharide binding and the prevention of bacterial growth [43].

The antibacterial activity of honey and chitosan has been previously reported in the literature [8,21,44]. The antibacterial action of the hydrogel dressing can be explained by the synergistic effect of chitosan and honey. When chitosan is dissolved in an acidic environment, the amino groups in the chains protonate into NH_3_^+^ and become cationic, allowing it to interact with various types of cell membranes. This positive charge is the main reason for the antimicrobial activity of chitosan. It interacts with the negatively charged cell membranes of the microorganisms, preventing their activity or resulting in cell death [45,46]. The antibacterial properties of honey depend on factors such as the osmotic effect due to the high sugar content and low pH [17]. A low pH results from the presence of organic acids in honey [22]. The presence of honey in a chitosan hydrogel can result in a synergistic antibacterial activity due to a lower pH and cationic charges. Accordingly, strong antibacterial activity was observed for the chitosan/PVA/honey hydrogel.

### 3.6. Release Study

**Figure 4:**
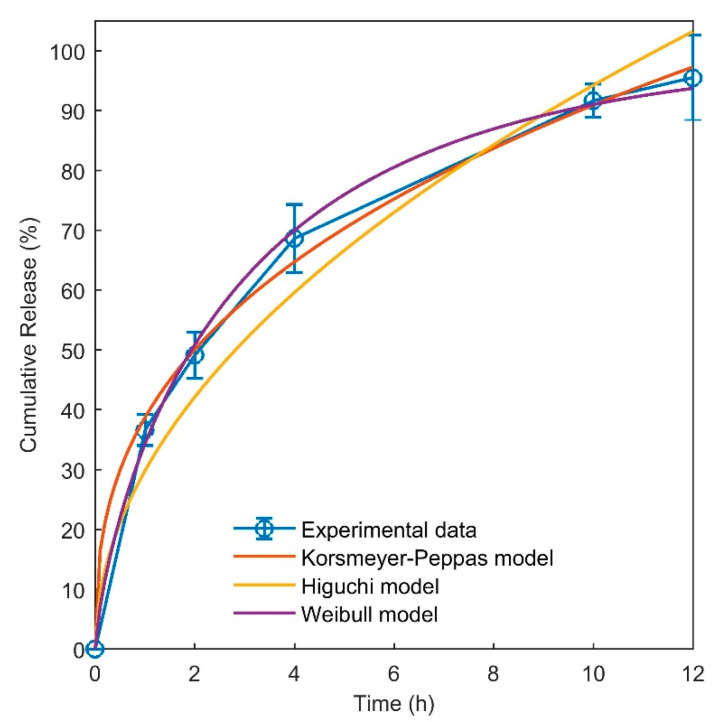
Release of allantoin from sample H3 at 37 °C and results of fitting the kinetic models to the release data.

The release of allantoin from the hydrogel sample H3 was studied to obtain data about the diffusion of allantoin from the hydrogels to the aqueous environment. The concentration–time curve shows that allantoin was gradually released from the hydrogel into the release medium within 12 h (Figure 4). After 1 h, around 36% of the total allantoin loaded in the films was released, and after 12 h, more than 95% of the drug was released. The hydrogel film starts swelling immediately after soaking in water. During this process, water molecules diffuse into the structure of the film. Due to the hydrophilic nature of PVA and chitosan and their favorable interactions with water, the polymer chains rearrange into more extended conformations and the free volumes are increased inside the hydrogel. Upon the entrance of water into the structure of the hydrogel, allantoin molecules are partially dissolved in the water and can readily diffuse through the pores created in the hydrogel due to the swelling phenomenon. As mentioned in the XRD and gel content results, the crosslinks formed by the freeze–thaw process have a physical nature due to the increased crystallinity of PVA. Immersion of the hydrogel film in the release medium results in the partial dissolution of the polymer chains. Thus, allantoin can also be released due to the dissolution of the hydrogel in the release medium, but as can be concluded from the in vitro degradation data, the timescale of degradation (within days) is much higher than the timescale of the release of allantoin (12 h). Accordingly, it can be concluded that the mechanism of allantoin release from the hydrogel sample is mainly diffusional. This conclusion is further examined by fitting the drug release models to the experimental data.

The results of the mathematical models (Equations (7–9)) fitted to the release data are presented in Figure 4 and Table 4. According to the results obtained, the release behavior of allantoin is best predicted by the Korsmeyer–Peppas and Weibull models, with a poorer fit to the Higuchi equation. The diffusion exponent “n” in the Korsmeyer–Peppas model demonstrates the mode of transport of the drug out of the matrix. In the case of a cylindrical geometry, a value of n lower than 0.45 implies Fickian diffusion [47]. Thus, the mathematical modeling of the drug release shows that the release of allantoin from the hydrogel followed the Fickian type of diffusional transport. The Weibull model also fitted well to the release data. The value obtained for the parameter b was 0.75, which corresponds to a parabolic curve which is the shape of the release curve (Figure 4). The parameter b in this model is also another indicator of Fickian diffusion [48]. The Higuchi model did not fit well to the release data. The reason is that this model assumes no significant alteration in the matrix when in contact with water, whereas the hydrogel matrices swell considerably during contact with water. Finally, the results of fitting the theoretical models to the experimental release data confirmed that the mechanism of allantoin release from the hydrogel is mainly diffusional.

### 3.7. Cytocompatibility

**Figure 5:**
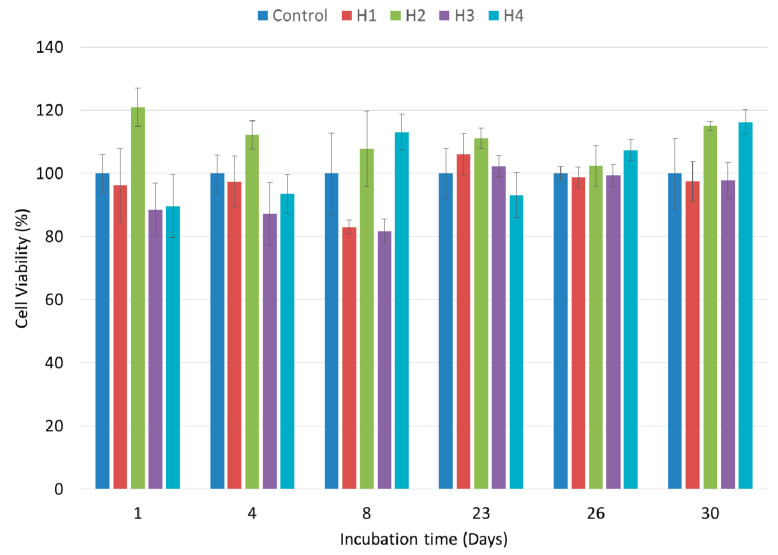
Cell viabilities of NHDF cells after 1, 4, 8, 23, 26 and 30 days of extract incubation obtained by indirect MTT assay method.

To evaluate the cytotoxicity and cell viability, all samples were tested using the indirect MTT assay method. The results for the percentage cell viability after incubation of the samples in serum-free culture medium for 1, 4, 8, 23, 26 and 30 days are shown in Figure 5. According to the ISO 10993-5 standard, it is crucial for the value of the in vitro cell viability of the cells to be above 70% in order to label the wound dressing as biocompatible. As can be observed, the cell viability values of all samples compared to the control were above 70%. These results confirm that the toxicity of the produced hydrogel samples is within a reasonable range, making them suitable for use as wound dressings or skin tissue engineering scaffolds [1,35]. Among the samples tested, the H2 hydrogel showed the highest cell viability at incubation times of 1,4 and 8 days, but its biocompatibility was close to that of the other samples at incubation times of 23, 26 and 30 days. The reason for this is the presence of honey in this sample. Due to the higher biocompatibility of sample H2 in the first week of culture, this sample was selected for the in vivo study. Sample H4, which also contained honey, showed high biocompatibility at time points 8, 26 and 30. The higher biocompatibility values of the H2 and H4 samples can be related to the presence of saccharides in honey which can act as nutrients for the growth of NHDF cells, increasing the biocompatibility.

MTT assays were performed within 1–7 days in many studies; however, we tested our samples over long-term incubation times. The results of this study suggest that the freeze–thaw method can be used as an effective and biocompatible method for crosslinking PVA/chitosan hydrogels, without any harmful effect on NHDF cells.

### 3.8. In Vivo Study

**Figure 6:**
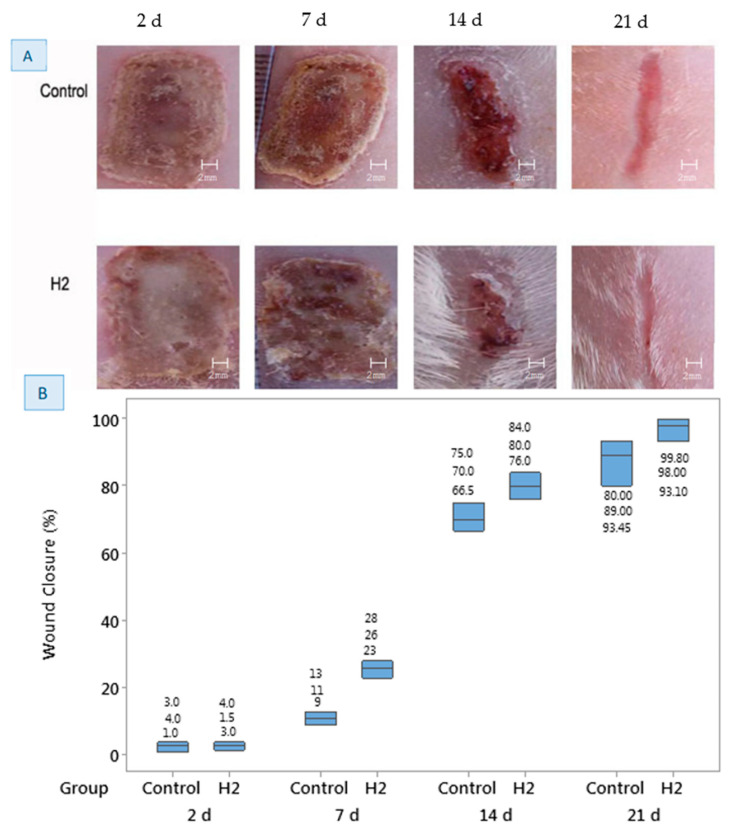
(**A**) Photographs of the wound repair for control and H2 hydrogel after 2, 7, 14 and 21 days. (**B**) Boxplot of the average area of the wound closed (%) at different healing times for control and H2 hydrogel.

In this study, the effect of the H2 sample on wound healing in a rat model was investigated. Images of the wound sites and the percentages of wound closure are shown in Figure 6A,B. On day 2, there was no particular difference in the closure of the wound between sample H2 and the control, as shown in Figure 6A. However, on day 7, the average wound contraction was 11% for the control group and ~25% for the H2 hydrogel. There is a large difference between the control sample and H2 after 7 days, which indicates the ability of the hydrogel to contract and heal the wound. On day 14, the percentage of the healed area for the control group was about 70%, while it was about 80% for the H2 sample, which is still higher than the control. Accelerated healing and wound contraction can also be seen from the image provided for the H2 sample on day 14 (Figure 6A). On day 21, as can be seen from Figure 6A, the closed area of the control sample was about 89%, while it was 98% for the H2 sample, which is much higher than the control. These results show that freeze–thawed chitosan/PVA/honey hydrogel accelerated the rate of wound healing in rats. In previous research, chitosan/Manuka honey bioactive wound dressings were evaluated for the healing of excisional wounds in a rat model [32]. The wound contraction obtained was 94% after 18 days of treatment with chitosan/Manuka honey wound dressing, which was significantly higher than the control (90%). Comparing the results of our study with this research, it may be concluded that the type of honey does not affect the wound contraction significantly, since our results for the common type of honey are comparable with those for Manuka honey.

The method used for crosslinking a hydrogel is very important for its application as a biomaterial. The effectiveness of chitosan/gelatin/honey hydrogels was reported in a previous study [22]; however, the crosslinking method was not mentioned. Gelatin was not present in our hydrogels. However, we showed that freeze–thawed chitosan/PVA/honey hydrogels can be used for wound healing without any concerns.

## 4. Conclusions

In this study, hydrogel wound dressings were prepared by freezing and thawing chitosan/PVA solutions containing honey and allantoin. Long-term biocompatibility studies showed that all the samples were biocompatible and non-toxic for 30 days. The hydrogel samples showed antibacterial activity against *E. coli* and *S. aureus* bacteria. Allantoin-containing samples showed stronger antibacterial activity against *S. aureus* compared to *E. coli*. The release of allantoin from the hydrogel took place via Fickian diffusion, and the Korsmeyer–Peppas and Weibull models fitted to the release data well. The hydrogel sample with chitosan/PVA/honey accelerated the wound healing in a rat model compared to the control group. The results of this study show that the freeze–thaw method can be used as an effective and non-toxic method for crosslinking of chitosan/PVA solutions without affecting the biocompatibility of the hydrogels.

## Figures and Tables

**Table 1:** Final composition and freeze–thaw cycles of the dry hydrogel films.

Sample	Chitosan/PVA (wt.%)	Honey (wt.%)	Allantoin (wt.%)	Freeze–Thaw Cycles
H0	100%	0	0	0
H1	100%	0	0	3
H2	95%	5%	0	3
H3	96%	0	4%	3
H4	91%	5%	4%	3

**Table 2:** Color parameters L*, a*, b*, whiteness index (*WI*) and yellowness index (*YI*) of the films in the dry state.

Sample	L*	a*	b*	*YI*	*WI*
H0	66.6 ± 0.1	−1.3 ± 0.3	0.9 ± 0.1	1.1 ± 0.5	60.1 ± 0.1
H1	66.2 ± 0.1	−1.1 ± 0.1	1.0 ± 0.1	1.5 ± 0.1	59.7 ± 0.1
H2	63.0 ± 0.2	−1.5 ± 0.1	21.9 ± 0.9	48.8 ± 2.0	53.6 ± 0.4
H3	66.2 ± 0.1	−2.1 ± 0.1	5.6 ± 0.1	12.0 ± 0.1	59.4 ± 0.1
H4	64.7 ± 0.1	−1.4 ± 0.2	14.3 ± 0.1	32.8 ± 0.1	56.6 ± 0.1

**Table 3:** Results for swelling ratio (in PBS pH = 7.4 at 37 °C), gel content (at 100 °C), mass loss, mechanical properties of the dry films (at 50 mm/min) and antibacterial efficiency of the hydrogels.

Sample	Swelling Ratio (%)	Gel Content (%)	Tensile Strength (MPa)	Elongation at Breaking (%)	Mass Loss (%)	Antibacterial Efficiency, E(%)	
						*E. coli*	*S. aureus*
H0	N/A	3.96 ± 2.1 ^A^^*^	21.8 ± 2.1 ^A^	190.5 ± 21.7 ^A^	25.3 ± 5.9	13.2 ± 1.4	3.3 ± 0.6
H1	476 ± 24 ^A^	4.58 ± 1.2 ^B^	19.8 ± 4.8 ^A^	141.43 ± 95.0 ^A^	12.6 ± 1.7	15.2 ± 2.1	27.4 ± 2.9
H2	421 ± 11 ^B^	8.72 ± 1.9 ^C^	16.7 ± 0.3 ^A,B^	421.6 ± 45.8 ^B^	52.5 ± 4.8	82.0 ± 4.8	48.1 ± 3.7
H3	468 ± 30 ^A^	5.72 ± 2.5 ^A^	19.3 ± 4.6 ^A,B^	156.7 ± 82.4 ^A^	36.7 ± 9.1	85.0 ± 5.3	71.1 ± 6.2
H4	324 ± 18 ^C^	3.82 ± 1.1 ^A^	10.6 ± 4.8 ^B^	241.0 ± 64.5 ^A^	57.7 ± 8.8	88.7 ± 5.9	77.0 ± 5.2

* The letters indicate grouping of the results obtained from the statistical analysis. Data with different letters are considered significantly different.

**Table 4:** Model fitting results for the release of allantoin from sample H3.

Model	Parameter	Value	Units
Korsmeyer–Peppas $\frac{M_{t}}{M_{0}}=K_{KP}t^{n}$	$K_{KP}$ n $R_{adjusted}^{2}$	0.38 ± 0.02 0.37 ± 0.04 0.99 ± 0.07	$S^{-n}$
Higuchi $\frac{M_{t}}{M_{0}}=K_{H}t^{0.5}$	$K_{H}$ $R_{adjusted}^{2}$	0.29 ± 0.04 0.96 ± 0.03	$S^{-0.5}$
Weibull $\frac{M_{t}}{M_{0}}=1-e^{\frac{-{\left(t-T\right)}^{b}}{a}}$	a b $R_{adjusted}^{2}$	2.38 ± 0.14 0.75 ± 0.05 0.99 ± 0.06	$S^{b}$

## Data Availability

The data presented in this study are available on request from the corresponding author.
